# Uterine fibroids in women diagnosed with acromegaly: a systematic review

**DOI:** 10.1007/s11154-024-09883-z

**Published:** 2024-04-26

**Authors:** Konrad Górski, Stanisław Zgliczyński, Maria Stelmachowska-Banaś, Izabella Czajka-Oraniec, Wojciech Zgliczyński, Michał Ciebiera, Magdalena Zgliczyńska

**Affiliations:** 1 Second Department of Obstetrics and Gynecology, Centre of Postgraduate Medical Education, Warsaw, Poland; 2 Department of Endocrinology, Centre of Postgraduate Medical Education, Warsaw, Poland; 3 Department of Internal Medicine and Endocrinology, Medical University of Warsaw, Warsaw, Poland; 4 Department of Obstetrics, Perinatology and Neonatology, Centre of Postgraduate Medical Education, Warsaw, Poland

**Keywords:** Acromegaly, Uterine fibroids, Leiomyomas, GH, IGF-1

## Abstract

The review discusses the relationship between acromegaly and uterine fibroids. It highlights variations in research methodologies and inconsistent findings, emphasizing the complex nature of fibroid development and the role of the somatotropic axis. Additionally, it addresses demographic factors and examines the potential impact of therapies on the risk and prevalence of uterine fibroids in individuals with acromegaly. We conducted an analysis of previously published literature that examined the repercussions of acromegaly on gynecological health in female cohorts, with specific attention directed towards elucidating the prevalence of uterine fibroids. We suggest that larger, more focused studies are needed to understand the specific impact of different treatments on the occurrence of gynecological issues in acromegaly patients. Additionally, our study emphasizes the importance of factors such as disease duration and treatment effectiveness. We hypothesize that a relationship between acromegaly and uterine fibroids may occur. However, it remains an area of ongoing research, with the need for larger, multi-center studies to draw more definitive conclusions.

## Introduction

Uterine fibroids are the most common benign tumors of the reproductive organ. Despite the increasingly available knowledge regarding the associated problems, it still seems to be an undervalued issue [1]. It is estimated that uterine fibroids occur in over 40% of women over 35 years of age. Research results vary, but the total incidence of fibroids in women of reproductive age is estimated at up to 70% in certain populations [2]. The pathophysiology of uterine fibroids, although still unknown, is multifactorial. The recognized factors that may modify the formation and growth of these tumors include age, black race, hormonal disorders or lifestyle-related factors, such as the level of physical activity or diet [3]. In most patients, the presence of uterine fibroids is asymptomatic, but in a certain group of patients these lesions cause problems that require a variety of medical interventions, such as pharmacological or surgical treatment [3–5]. The most common symptoms associated with fibroids include: anemia secondary to heavy menstrual bleeding, pain within the lesser pelvis, painful menstruation. Fibroids may also contribute to infertility or pregnancy-related disorders, but these just constitute some of the wide range of symptoms [6]. Importantly, all the symptoms may significantly affect the quality of patients’ lives [7].

As these are hormone-dependent gynecological tumors, the development of uterine fibroids depends on sex steroids, i.e., estrogens. Moreover, progesterone is becoming a more common focus of researchers discussing the conditions underlying the development of these tumors [8]. Recent research has highlighted the potential contribution of progesterone to the pathophysiology of uterine fibroids. Progesterone may interact with growth factors, such as epidermal growth factor (EGF), transforming growth factor beta-3 (TGF-beta3), and insulin-like growth factor-1 (IGF-1), thereby promoting the proliferation and survival of fibroids. Another possible action is linked to progesterone-related progenitor stem cell activity. Furthermore, progesterone may be responsible for the regulation of angiogenesis. At the fibroma level, the number of progesterone receptors was found to be elevated. In summary, progesterone appears to play a pivotal role in the growth of uterine fibroids [9, 10].

The GH (growth hormone)– IGF-1 (insulin-like growth factor 1) axis plays a key role in the regulation of cell growth, cell differentiation and death, and, what follows, in the development and metabolism of the entire human body. IGF-1 has anti-apoptotic properties– it stimulates the cell cycle and cell division. It is also a promoter of angiogenesis [11]. Some authors indicated the important role of IGF-1 and GH in the pathogenesis of tumors. It is believed that this axis is able to move already transformed cells through subsequent phases of the cell cycle. Viral and cellular oncogenes require an intact IGF-1 signaling pathway to induce transformation [10]. Influencing this axis provides certain possibilities of oncological therapy [12, 13].

According to some authors, IGF-1 may be a factor that promotes the proliferation of uterine fibroid cells. For example, IGF-1 receptors were shown to be overexpressed in fibroid tissues, compared to the myometrium [14]. To date, the participation of increased IGF-1 concentration has been suggested in the pathophysiology of the formation of the tumors [15]. An increased expression of the IGF-1 receptors in the fibroid tissue may also be associated with the promotion of angiogenesis and partially underlie the pathophysiology of heavy menstrual bleeding in patients with uterine fibroids [16].

Acromegaly is an example of a disease characterized by an increased concentration of GH and IGF-1. The manifestations of acromegaly are caused by an increased GH secretion by pituitary tumor and, as a consequence, increased IGF-1 levels. It was also confirmed that the severity of disease symptoms as well as the frequency of complications and GH concentrations were positively correlated with both the duration of the disease and the size of the pituitary tumor [17]. Acromegaly is a rare disease with an overall incidence ranging between 2.8 and 13.7 cases per 100,000. The incidence is 0.2–1.1 cases/year/100,000 people. The peak of acromegaly diagnoses is noted in the 5th decade of life and the average age at diagnosis is 40.5–47 years. The diagnosis is most often made about 5 to 10 years after the onset of the first symptoms [18]. However, recent research has suggested a higher prevalence than previously assumed, i.e., 85/10^6^ [19, 20].

Regrettably, despite the growing awareness of physicians and patients, the diagnosis of acromegaly is still delayed even though the manifestations of the disease are rather characteristic [21]. These mostly include changes in the external appearance, such as the enlargement of the hands, feet, the facial part of the cranium, coarse facial features, weight gain with changes in body composition characterized by ectopic lipid deposition in muscles and reduced visceral adiposity and intrahepatic lipid, as well as organomegaly, soft tissue or bone enlargement [22, 23]. It was also demonstrated that the disease and its complications significantly reduced the quality of patients’ lives [24].

An important role in the diagnosis of acromegaly is played by the above-mentioned growth factor, i.e., IGF-1. The measurement of serum IGF-1 levels is crucial in the diagnosis of acromegaly in patients with suspected disease based on clinical symptoms along with the oral glucose toleration test-induced GH suppression as a confirmation test [25]. IGF-1 is also an indicator of acromegaly activity; its serum level, in correlation with GH level is useful in monitoring disease progression [26, 27].

It is estimated that untreated acromegaly may reduce the average survival time by about 10 years. Early diagnosis and treatment of the disease is crucial due to the numerous complications that untreated acromegaly may lead to [28]. The most dangerous of those complications, apart from cardiovascular and respiratory issues, include the increased occurrence of both benign and malignant tumors. Therefore, it is important to note that cancer became the leading cause of death in the group of patients with acromegaly in the last decades [29]. 

The occurrence of tumors in patients with acromegaly has been the subject of numerous studies. Data from previous studies are strongly inconclusive in this area. Some authors confirmed a higher incidence of neoplasms in patients with acromegaly [30–33], while others showed that the overall risk of cancer in patients with acromegaly might not differ at all from the risk in the population [34]. A four-fold increase in the incidence of kidney and bladder cancer was demonstrated in a group of patients with acromegaly [35]. Moreover, an increase in the incidence of colorectal cancer was also found, which was confirmed in other studies as well [36]. Interestingly, no increase in tumor-related mortality was noted. Conversely, another study showed no increased incidence of thyroid, respiratory, brain, breast or prostate tumors in patients with acromegaly [35]. However, the available data are inconclusive, as other studies suggested an increased incidence of thyroid diseases in patients with acromegaly, including the aforementioned thyroid cancer [37]. Some authors suggested only a slightly elevated overall risk of cancer in patients with acromegaly [38]. Nevertheless, according to current guidelines, screening colonoscopy at the diagnosis is recommended [39].

The potential impact of the GH-IGF-1 axis on the formation of fibroids, which was demonstrated in basic research [14], determines the need to assess the frequency of this pathology in a group of patients with acromegaly. The authors of one of few available studies on uterine fibroids in patients with acromegaly indicated a possible association of acromegaly with an increased risk of developing this pathology. It should be emphasized that the study was conducted in a group of only 25 patients [40]. However, contrary to this hypothesis, the results of some studies confirmed no such relationship [41]. The data seem to be of limited strength, even considering the fact that acromegaly is a rare disease. The hypothesis regarding the relationship between fibroids and acromegaly was not verified in any subsequent study or in a larger population. Therefore, such views remain unchanged and are passed on to subsequent generations of gynecologists.

The relationship between acromegaly and tumor formation in the reproductive system remains ambiguous. Currently available research results are insufficient to draw clear conclusions and leave room for further research. In this paper, we aimed to examine the available data concerning the relationship between the GH–IGF-1 axis and uterine fibroids.

## Materials and methods

Preferred Reporting Items for Systematic Reviews and Meta-Analyses Statement (PRISMA) were followed when designing and developing the review [42]. Three databases: the Cochrane Library, PubMed/MEDLINE and Scopus were used to obtain manuscripts. We did not take account of the publication time criteria. The last search was made on 15 March, 2023. Our search strategy is shown in Table 1.

A total of 1998 articles were retrieved. We used the automatic search function in EndNote X9 (Clarivate Analytics, London, UK) and 460 duplicates were removed. Another 96 repeated articles were manually deleted by SZ. The remaining 1442 manuscripts were screened by the authors (SZ and KG). We present the inclusion and exclusion criteria of our review in Table 2.

**Fig. 1:**
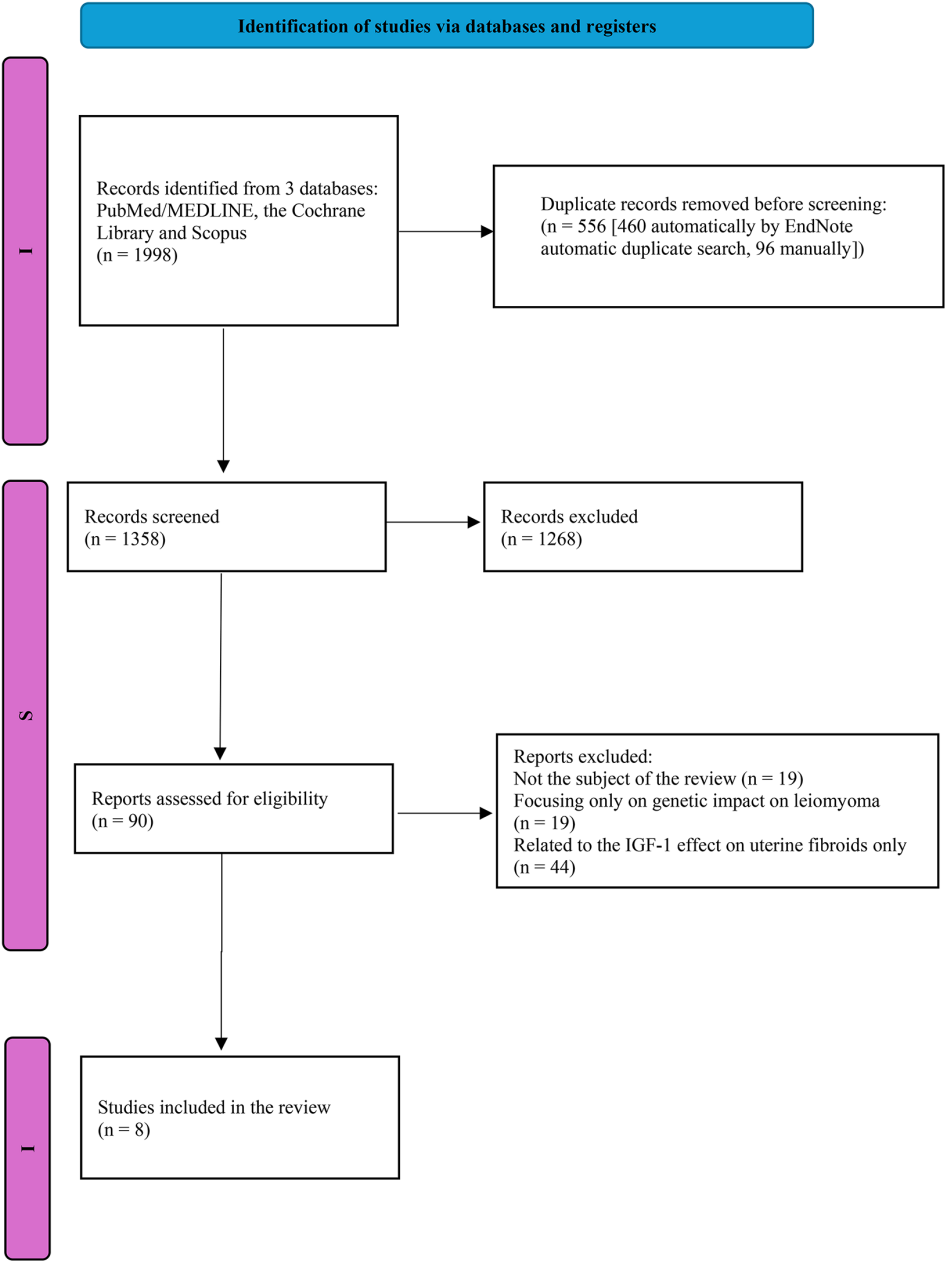
PRISMA 2020 diagram. *From* Page MJ, McKenzie JE, Bossuyt PM, Boutron I, Hoffmann TC, Mulrow CD, et al. The PRISMA 2020 statement: an updated guideline for reporting systematic reviews. BMJ 2021;372:n71. doi: 10.1136/bmj.n71

Subsequently, the authors of this review (SZ and KG) carried out eligibility assessment independently. Any disagreements were discussed by the authors separately. We present the selection details in the PRISMA Flow Diagram (Fig. 1).

In order to collect specific information about: the authors, nationality, date, type, aim of the study and results, we used a specially designed data extraction sheet. To increase the accuracy of our search a double-checked correction was performed by MC. The results are presented on the adjusted PRISMA 2020 diagram.

**Table 1:** Databases and the search strategy

Database	Number of results	Search strategy
PubMed	609	(“Acromegaly“[Mesh] OR acromegal*[tiab] OR “Growth Hormone-Secreting Pituitary Adenoma“[Mesh] OR “Somatomedins“[Mesh] OR somatomedin*[tiab] OR (insulin[tiab] AND like[tiab] AND growth[tiab] AND factor[tiab]) OR IGF[tiab] OR IGFR*[tiab] OR “Growth Hormone“[Mesh] OR (growth[tiab] AND hormone[tiab]) OR somatotropin*[tiab]) AND (“Leiomyoma”[Mesh] OR myom*[tiab] OR leiomyom*[tiab] OR fibromyom*[tiab] OR (uterine[tiab] AND fibroid*[tiab]) OR (uterine[tiab] AND fibrom*[tiab]))
Scopus	1340	TITLE-ABS-KEY (acromegal* OR (growth AND hormone AND secreting AND pituitary AND adenoma*) OR somatomedin* OR (insulin AND like AND growth AND factor*) OR igf* OR igfr* OR (growth AND hormone) OR somatotropin*) AND TITLE-ABS-KEY (myom* OR leiomyom* OR fibromyom* OR (uterine AND fibroid*) OR (uterine AND fibrom*)) AND (LIMIT-TO (LANGUAGE, “English”))
Cochrane	49	#1 MeSH descriptor: [Acromegaly] explode all trees #2 acromegal* #3 MeSH descriptor: [Growth Hormone-Secreting Pituitary Adenoma] explode all trees #4 MeSH descriptor: [Somatomedins] explode all trees #5 somatomedin* OR (insulin AND like AND growth AND factor) OR IGF* OR IGFR* #6 MeSH descriptor: [Growth Hormone] explode all trees #7 (growth AND hormone) OR somatotropin* #8 MeSH descriptor: [Leiomyoma] explode all trees #9 myom* OR leiomyom* OR fibromyom* OR (uterine AND fibroid*) OR (uterine AND fibrom*) #10 (#1 OR #2 OR #3 OR #4 OR #5 OR #6 OR #7) AND (#8 OR #9) And Trials

**Table 2:** Inclusion and exclusion criteria

Inclusion Criteria	Exclusion Criteria
All types of original articles in the field of uterine fibroids in acromegalic patients	Editorials, textbooks reviews, conference papers and abstracts, case series, case reports, familial case reports
Published in peer-reviewed scientific journals	Published in journals other than peer-reviewed ones
Full text articles available in English	Languages other than English, only abstracts available in English
No restriction in the time of publication	-
Human studies	Animal studies

**Table 3:** Results The above described systematic search allowed authors to retrieve 8 eligible studies that originated from 6 different countries, published in the period of 1991–2023. Table 3 contains basic data about the selected studies and the main results and conclusions (Table 3)

First author, year of publication, country of origin	Study period	Number of women with acromegaly considered in terms of uterine fibroids	Age [years]	Acromegaly– data on disease	Acromegaly– data on treatment	Incidence of uterine fibroids in the acromegalic women subpopulation	Other main results and/or author conclusions on uterine fibroids
*Barzilay et al. 1991* *United States* [31]	1957–1988	43	Median 37, range 16–70 (for both men and women)	∙ Median age at diagnosis– 37 years ∙ Median time from onset– 5 years (for both men and women)	∙ Surgery– 33% ∙ Pharmacotherapy– 3% ∙ Radiotherapy– 60% (for both men and women)	12%	The incidence of uterine fibroids in acromegalic patients was higher compared to the control group (non-functioning tumors or prolactinomas)
*Cohen et al. 1998* *Israel* [40]	1967–1992	25 (16 gynecologically examined)	Median 43, range 28–74	∙ Median age at diagnosis– 43 years ∙ Median diagnosis latency– 6.5 years	∙ Surgery– 40% ∙ Pharmacotherapy– 20% ∙ Radiotherapy– 36%	81%	Authors concluded that uterine fibroids should be considered as the inclusion of GH excess as a feature of organomegaly associated with acromegaly
*Kaltsas et al. 2000* *United Kingdom* [43]	No data	15	Median 44.5, range 26–50	No data	No data	33%	Authors concluded that vigilance was necessary in seeking the presence of leiomyomas in women with acromegaly, particularly in the older age group
*Bałdys-Waligórska et al. 2010* *Poland* [44]	1983–2008	71	Mean 52 (for both men and women)	∙ Microadenoma– 32%; Macroadenoma– 68% ∙ Mean observation period– 9.4 years ∙ Mean period of uncontrolled disease 6.4 years	No data	12% (and/or polyps)	-
*Dogansen et al. 2018* *Turkey* [45]	No data	47	No data	∙ Mean age at diagnosis– 34 years ∙ Microadenoma 5; Macroadenoma 42 ∙ Mean disease duration 3.6 years	No data	53% (and/or polyps)	-
*Matyjaszek-Matuszek et al. 2018* *Poland* [46]	2004–2015	46	Range 24–75 (for both men and women)	∙ Mean age at the onset of the first symptoms– 41 ∙ Mean age at diagnosis– 49 (for both men and women)	∙ Surgery– 72% ∙ Pharmacotherapy– 28% (for both men and women)	17%	-
*Costa et al. 2021* *Italy* [47]	2000–2019	122	Median 59, range 32–83 (for both men and women)	∙ Mean disease duration 16.8 years ∙ Mean diagnostic latency 3.4 years (for both men and women)	∙ Surgery– 74% ∙ Pharmacotherapy– 76% ∙ Radiotherapy– 18% ∙ No treatment– 2% (for both men and women)	Benign lesion (colon polyposis or/and multinodular goiter or/and uterine fibromatosis) in 33%	-
*Pirchio et al. 2023* *Italy* [48]	2005–2019	50	At diagnosis Mean 38 At disease control Mean 45	∙ The age at the onset of symptoms– 17–45 ∙ Diagnosis latency– mean 7 years, range 6 months– 20 years ∙ Microadenoma 24%; Macroadenoma 76% ∙ Active disease duration: mean 7, range 1–31 years	∙ Surgery– 78% ∙ Pharmacotherapy– 94%	∙ At persumed disease onset– 18% ∙ At diagnosis– 39% + 4 patients had undergone hysterecomy for uterine leiomyoma before diagnosis ∙ At disease control– 30%	Compared to the presumed disease onset, the prevalence of uterine leiomyoma was significantly higher There was no significant change in the prevalence of uterine leiomyoma when compared diagnosis to disease control

The total number of patients in the studies included in the review was accounted for 419, and the largest group was examined by Costa et al., i.e., 122 women diagnosed with acromegaly [47]. Most studies took account of a wide range of patient ages, both pre- and postmenopausal. Acromegaly was also characterized in different ways, but studies that reported the age at diagnosis yielded similar results. The treatment of acromegaly also varied. There is a clear trend towards a more frequent use of surgery in studies from the last decade compared to older ones. Most importantly, the incidence of uterine fibroids varied significantly– from 12% to as much as 81% of women with acromegaly included in individual studies.

## Discussion

As presented in the results section, the conclusions drawn from the presented studies are significantly different from one another. There may be several factors contributing to this, which will be discussed below. First of all, the studies differed significantly in methodology. They concerned different nationalities and, therefore, ethnic groups, and women with acromegaly of different ages. These two main factors undoubtedly influenced the results regardless of the diagnosis and severity of acromegaly. Moreover, from 1957 to 2023 there was a revolution in the treatment of acromegaly (the spread of surgery, new pharmacological options) and the control of the disease undoubtedly improved.

It is known that the formation of fibroids is a multidimensional and not fully elucidated process. Therefore, we should also consider other factors that are not directly related to acromegaly but promote the formation of uterine fibroids, including genetic and environmental factors, or obesity, and other factors that modify the course of the disease. The conclusion drawn from those analyses is that elevated concentrations of IGF and acromegaly may not be considered as unquestionable factors of an increased risk of the development and growth of uterine fibroids.

The vast majority of studies had no control group, so it is difficult to draw conclusions concerning the increased incidence of uterine fibroids. However, the study results that stand out are those presented by Cohen et al. on the Israeli population of patients [40]. This relationship could also result from the genetic determinants of this group of patients. Geographical variability and the resultant difference in the populations of patients with acromegaly may also play a role. As regards genetic factors, it may be worth considering an increased incidence of neoplastic disorders in specific endogamous communities, e.g., in the population of Ashkenazi Jews, in whom studies confirmed an increased incidence of tumors such as hereditary breast and ovarian cancer, Lynch syndrome, or Kaposi sarcoma [49–51]. However, in case of uterine fibroids, it is not the conclusion resulting from research, but one of hypotheses.

An important issue affecting the quality of the conclusions drawn is related to the fact that some studies included general conclusions instead of focusing on individual pathologies of the reproductive system in women. Dogansen et al. did not analyze the diagnosis of endometrial polyps and uterine fibroids separately [45]. Similarly, the incidence of uterine fibroids was not separately assessed by Costa et al. However, attention was paid to the generally increased incidence of benign proliferative lesions, which, apart from uterine fibroids, included thyroid tumors and colorectal polyps [47]. Therefore, the actual incidence of uterine fibroids is simply difficult to determine in numerous papers.

Some of them mostly concentrated on demographic issues. However, our attention was also drawn to the issue of the impact of certain therapies on the possible risk of fibroids or the course of the disease. At this point, it should be considered how the treatment of the cause of the disease affects the incidence of uterine fibroids in the population of women with acromegaly. As already mentioned, currently, surgical treatment of pituitary tumor remains the gold standard [52]. Only some authors tackled this issue. Pirchio et al. differentiated groups of patients undergoing surgical, pharmacological and combined therapy. Similarly, studies by Barzilay et al., Cohen et al., Matyjaszek et al., and Costa et al. comprised individual methods of acromegaly treatment. The remaining authors took no account of the impact of this factor on the complications of the disease. It should also be noted that authors of some papers which did not focus on women’s health problems did not analyze the data for the population of women and men separately. Therefore, the collected data made it difficult to refer to them in the context of the occurrence of diseases of the female reproductive system, including uterine leiomyoma.

Overall, there is room in subsequent publications for a thorough analysis of the impact of the type of therapy on the course of the disease and the incidence of reproductive system diseases in women suffering from acromegaly. It would be useful to study a larger group of patients, focusing on the occurrence of gynecological problems, taking account of the course of the disease, its duration and the type of treatment implemented. However, it is difficult due to the rare occurrence of the disease, which makes it much harder to draw conclusions and determine the relationship between the occurrence of acromegaly and gynecological diseases. It is possible to observe patients under the care of multidisciplinary reference centers for many years in few cases only. Moreover, registers, such as the Register of Acromegaly Patients in Poland, are kept to facilitate access to data, since it is the best way to expand knowledge about rare diseases.

From the perspective of public health, the fertility of patients with acromegaly and the impact of treatment on the cause of the disease are other important issues. The most common disorders in patients with acromegaly include menstruation and ovulation issues [48]. A study by Dogansen et al. revealed the occurrence of such disorders in 62% of patients [45]. Uterine fibroids probably play a lesser role in the infertility of women with acromegaly. It should be noted that Pirchio et al. demonstrated that good disease control was associated with a reduction in the frequency of gynecological complications. The study showed a reduction in the incidence of PCOS, ovarian cysts, and menstrual disorders, but not a significant reduction in the incidence of uterine fibroids in the treatment group [48].

It should also be noted that studies by Cohen et al. and Kaltsas et al. did not take account of a very important factor in the occurrence of disease complications, i.e., the duration of the disease. The duration of untreated disease and the mode of treatment used, followed by the control of its effectiveness, are also important. It was demonstrated that the number and severity of complications of the disease were much higher in patients with a long history of the disease than in patients diagnosed and treated early [53]. The above conclusions also stay in line with those of other studies confirming that good disease control increased the quality of life and life expectancy in patients with acromegaly [17, 54]. This emphasizes the role of patient-tailored, multidisciplinary therapy for patients with acromegaly, which was also suggested in available literature [55].

## Conclusions

Overall, uterine fibroids occurring in the course of acromegaly are a subject that still requires enhancing in terms of medical knowledge. Furthermore, available data pertaining to the impact of acromegaly on uterine fibroid incidence are insufficient, indicating a pressing need for further investigation.

The requisite for expansive cohorts, drawn from diverse geographic locations and medical facilities, presents a notable challenge due to the rarity of acromegaly. This underscores the necessity for concerted efforts in research endeavours. Multidisciplinary and multicenter approaches are imperative to ensure comprehensive care for individuals with acromegaly who struggle with a multitude of complications of the disease, including uterine fibroids.

## Data Availability

Data available from the authors upon reasonable request.
